# One vs. two non-symbolic numerical systems? Looking to the ATOM theory for clues to the mystery

**DOI:** 10.3389/fnhum.2013.00073

**Published:** 2013-03-13

**Authors:** Christian Agrillo

**Affiliations:** Department of General Psychology, University of PadovaPadova, Italy

In this interesting paper, Hyde (2011) summarized the current debate surrounding non-symbolic numerical abilities in human and non-human species. Evidence collected in the fields of cognitive, developmental, and comparative psychology supports the idea of two different numerical systems that exist in the absence of language: a precise object tracking system (OTS) for small numbers—which is supposed to support the accurate enumeration of small sets (≤4) without serial counting (subitizing)—and an approximate number system (ANS) for larger numbers based on analog magnitudes. The lack of a ratio effect is considered to be the main signature that allows experimental differentiation of the OTS from the ANS (Feigenson et al., 2004; Agrillo et al., 2012): in a few words, our accuracy is similar when we are required to discriminate one vs. four or three vs. four items (due to the OTS), whereas we are much more accurate in discriminating 6 from 24 than 18 from 24 items (due to the ANS).

However, while the existence of the ANS is generally accepted, researchers tend to disagree on whether a distinct, precise system really operates within 3–4 units (Ansari et al., 2007; Hyde and Spelke, 2009; vanMarle and Wynn, 2009; Agrillo et al., 2012). To explain the inconsistency reported in the literature, Hyde hypothesized that the ANS may be recruited to represent small numbers and that the limits of attentional resources and working memory would play a key role in determining which of the two systems would be employed in the small number range. Several lines of evidence indeed support his view. A very recent study, however, has provided new insights into the issue, showing that attention and working memory are not the only factors and prompting the inclusion of expertise in magnitude estimation in the current discussion.

To better understand the problem at its core, it is important to take a page from the ATOM theory (“a theory of magnitude”) that Walsh (2003) advanced. According to the author, a common magnitude system located mainly in the parietal lobe would process a non-symbolic estimation of time, space, and numbers. One potential prediction of this theory would be that increased abilities in one domain should correlate with increased abilities in another. In this sense, experts in one domain (i.e., time estimation) should exhibit better performance in tasks that are not directly related to their domain of expertise (i.e., spatial or numerical estimation) given the existence of a singular cognitive system applied to these three magnitudes. To test this hypothesis, Agrillo and Piffer (2012) compared the performance of musicians and non-musicians in temporal, spatial, and numerical discrimination tasks where verbal processing of the stimuli was experimentally prevented. Musicians proved to be better able to discriminate not only temporal dimensions but also spatial and numerical dimensions, supporting the idea of a general magnitude system.

In particular, a different pattern of data was observed within and beyond the subitizing range. Musicians were more accurate in the large number range, suggesting that musical training might have led to an increased precision of the ANS. However, musicians' performance in the subitizing range showed ratio dependence—the typical signature of the ANS—as their accuracy decreased when the numerical ratio between the small and large numbers increased. The most likely explanation is that musicians have adopted the trained ANS even in the small number range. As a control test showed that the two groups did not differ in attention and working memory, the activation of the ANS in the subitizing range seems to be due to the different levels of expertise. It is interesting to note that expertise did not improve the OTS. After all, the OTS is believed to afford numerical comparison only indirectly through one-to-one correspondence (Trick and Pylyshyn, 1994), and therefore, it might not appear surprising that the OTS is not included in the common system for time, space, and numbers. In this sense, musical training could not improve the OTS.

It is currently unknown as to how exactly expertise contributes to increased use of the ANS in the small number range. One hypothesis is that utilizing the ANS might not be an effect of expertise so much as an effect of acuity, which expertise may influence. Conversely, it might be that experts' regular use of the ANS makes it more likely to be engaged regardless of how accurate the ANS really is. The former hypothesis can actually be tested by correlating the slope of the ratio effect in the small number range with the overall accuracy of participants. As no correlation was found (*p* > 0.05) in the sample that Agrillo and Piffer (2012) tested, the latter hypothesis seems to be more likely: expert musicians may recruit the trained ANS regardless of its accuracy. To assess whether the same conclusions can be generalized to other data sets, such an analysis should be encouraged in studies in which participants have different levels of expertise.

The conclusions of this study evidently must be analyzed not only within the theoretical framework of ATOM but also within the debate surrounding non-symbolic numerical abilities: expertise in temporal, spatial, or numerical domains is yet another contextual factor that might determine the activation of the ANS in the small number range. It is known that expertise may lead to exceptional performance and may determine the consistent modification of neuro-cognitive systems, although not necessarily those related to the main domain of expertise (Gauthier et al., 2000). As far as I can see, however, this variable has seldom been taken into account in the literature on this topic.

Of course, further studies are necessary to shed light on the exact role of extensive training in magnitude estimation. With respect to this point, DeWind and Brannon (2012) have recently administered to participants a simple numerical training and discovered an improved ability in a numerical but not in a spatial task. This is in line with a weaker version of ATOM according to which space, time, and numbers would be at least partially differentiated. It is worth noting that the training that DeWind and Brannon (2012) set up lasted 2 weeks, well below the level of expertise that Agrillo and Piffer (2012) investigated in the temporal domain (all musicians had conservatory of music degrees and had played musical instruments for at least 12 years). It is possible that only long-term training can shape the common magnitude system in the way that Agrillo and Piffer (2012) hypothesized. Also, a large debate exists as to whether mathematical achievement is positively correlated to the precision of our ANS (Halberda et al., 2008; Ranzini, 2010; Libertus et al., 2012) or not (Castronovo and Göbel, 2012). Regardless of these questions, the ratio dependence that musicians exhibit in the small number range (Agrillo and Piffer, 2012) is worth noting and suggests the possibility that expertise in magnitude estimation might be sufficient to generate increased reliance on the ANS, even in the absence of the memory and attentional demands that Hyde (2011) noted.

Many questions still persist, but one thing is certain: The “trinity” of magnitudes that Walsh (2003) advanced seems to have tripled the problems for scientists who work in just one of the abovementioned fields (temporal, spatial, and numerical cognition).
